# Future perspectives in mass spectrometry of plant lipids

**DOI:** 10.1007/s00425-026-04981-5

**Published:** 2026-03-24

**Authors:** Katharina Gutbrod, Peter Dörmann

**Affiliations:** Institute of Molecular Physiology and Biotechnology of Plants (IMBIO), University of Bonn, Karlrobert-Kreiten-Strasse 13, 53115 Bonn, Germany

**Keywords:** Biosynthesis, Lipidomics, Mass spectrometry, Organelle, Subcellular distribution, Transport

## Abstract

**Main conclusion:**

Important topics of plant lipidomic research include the standardization of protocols for quantification, and the analysis of subcellular distribution of common and unusual lipids, both in Arabidopsis and non-model species.

**Abstract:**

Plant lipid research has seen a tremendous progress in the last decades, particularly in the area of lipid analytics by mass spectrometry. This includes the characterization of the different lipid classes involved in the establishment of the membrane bilayer, in carbon storage, and signaling. Advances in mass spectrometry have transformed the landscape of plant lipid research, enabling large scale studies of complex lipids at the level of individual molecular species, with minimal efforts of sample preparation. Lipidomic technologies employ targeted approaches to analyze known lipid molecular species as well as non-targeted methods to identify lipids that accumulate differentially in specific sample sets. Lipid quantification requires the availability of appropriate standards and highly sensitive methods of mass spectrometry. Additional technologies have been developed to study the spatial distribution of lipids in plant tissues, as well as to identify and characterize unusual lipids in plant cells. Finally, the large amounts of data generated in plant lipid research require sophisticated databases that connect the ‘omics’ data with data on growth, development, and adaptive responses to stress conditions at the tissue, cellular, and subcellular levels.

## Introduction

Lipids play important roles in human nutrition, medical applications, biotechnology, and as a resource for biofuels. Research on lipids started in the eighteenth century with the isolation of fatty acids, phosphoglycerolipids, sphingolipids, and sterol lipids, and the subsequent elucidation of their roles in animal, bacterial, fungal, and plant metabolism. Lipid research has undergone significant advancement in the recent decades through the development of new analytical tools, in particular mass spectrometry-based technologies. Technical advances in lipid analysis along with the elucidation of the lipid biosynthetic pathways in the model plant *Arabidopsis thaliana* have resulted in a comprehensive understanding of the regulation of lipid synthesis and lipid function in plants. Lipid metabolism in Arabidopsis is closely related to that of other Brassicaceae species, such as oilseed rape (*Brassica napus*). This is due to the presence of both the prokaryotic (chloroplast-localized) and eukaryotic (ER-localized) pathways for lipid synthesis in these two species. In contrast, other species, such as pea (*Pisum sativum*), lack the prokaryotic lipid pathway. Furthermore, the lipid metabolism of plant seeds can vary considerably between different species, due to the accumulation of various unusual fatty acids in the seed oil of different plants (Ohlrogge and Browse 1995). Plant lipidomics is defined as the comprehensive analysis of lipids in plant tissues, employing MS-based techniques. Compared with mammalian lipidomes, plant lipids exhibit a unique structural diversity, because plants contain complex glycerolipids (phosphoglycerolipids, glycoglycerolipids), sphingolipids (glucosylceramides, glycosylated inositol phosphoceramides, GIPC), surface lipids (cuticular waxes, cutin and suberin polymers), and isoprenoid lipids (sterol lipids: sitosterol, stigmasterol, campesterol, their esters, glycosides and acylated glycosides; carotenoids; quinone lipids including tocopherol, phylloquinone, plastoquinone, ubiquinone). Plant lipids are known to contain a variety of unusual structural features, including unusual fatty acids, long chain bases, or phytosterols, which are not found in animal lipids. Lipids are known to play pivotal roles in the metabolism and physiology of plants. For example, polar lipids form the plasma membrane and the membranes of the cellular compartments, storage lipids serve as reservoir for carbon and energy, redox-active lipids mediate electron transfer in photosynthesis and oxidative phosphorylation, and signaling lipids are important cellular messengers. In addition, different phytohormones including abscisic acid, gibberellic acid, oxylipins (e.g., jasmonic acid, oxophytodienoic acid), brassinosteroids, and strigolactones are derived from lipid metabolism.

Different technologies have been developed for the analysis of the vast diversity of plant lipid classes. Historically, lipid analyses were carried out using liquid–liquid extraction, column chromatography, thin-layer chromatography (TLC), and gas chromatography (GC), frequently in conjunction with each other. The lipid structures were elucidated down to the position and configuration of double bonds, chemical modifications (hydroxyl group, methyl group) of acyl chains, and the *sn* distribution of acyl groups on glycerolipids. Over the past 3 decades, MS-based lipidomic techniques have been developed and employed for the analysis of animal and plant lipids (Welti et al. 2002; Han and Gross 2005). Lipidomic techniques encompass targeted (quantification of predefined lipids) and non-targeted (discovery of unknown lipids) workflows, supported by standardized nomenclature, advanced ionization strategies, and expanded spectral libraries. The following sections summarize key methodological developments, instrumentation, quantification strategies, and future challenges in plant lipidomics.

## Mass spectrometry platforms and ionization techniques

The field of plant lipidomics is predominantly reliant upon three MS platforms, each of which with distinct advantages (Table 1). Triple quadrupole (QQQ) instruments predominate in targeted analyses due to their high sensitivity and multiplexing capabilities, while quadrupole-time-of-flight (QTOF) and Orbitrap systems are particularly effective in non-targeted workflows, offering high-resolution MS/MS for structural elucidation. All instruments can be used for tandem MS experiments: MS1 refers to the first stage, in which intact precursor ions are measured to determine their mass-to-charge ratios; MS2 refers to the second stage, which involves the fragmentation of a selected precursor ion and the analysis of the resulting product ions for structural information.

**Table 1:** Common MS platforms in plant lipidomics

Separation method	MS type	Key applications	Characteristics	References
None (DI)	QTOF/Orbitrap	High-throughput lipid profiling	High resolution, low scan rates	Gutbrod et al. (2021)
None (DI)	QQQ	Targeted quantification	Low resolution, high sensitivity	Welti et al. (2002)
LC	QQQ	Targeted quantification	Low resolution, high sensitivity	Jouhet et al. (2017), Herrfurth et al. (2021)
LC	QTOF/Orbitrap	Non-targeted analyses	High resolution, low scan rates, low sensitivity	Okazaki et al. (2013a)

*DI* direct infusion, *LC* liquid chromatography, *QQQ* triple quadrupole, *QTOF* quadrupole time of flight

Electrospray ionization (ESI) is the most widely used ionization technique in plant lipidomics. It is compatible with liquid chromatography–mass spectrometry (LC–MS) and direct infusion-mass spectrometry (DI-MS or shotgun lipidomics). ESI can efficiently ionize polar lipids (e.g., phosphoglycerolipids, glycoglycerolipids), but less efficiently non-polar lipids. In addition, ESI can be susceptible to ion suppression in complex matrices. It is possible to overcome these drawbacks by enriching low abundant lipids prior to MS analysis (for example with TLC or solid-phase extraction, SPE), or by LC–MS. Alternative ionization techniques are matrix-assisted laser desorption/ionization (MALDI) and desorption electrospray ionization (DESI) (see below), and atmospheric pressure chemical ionization (APCI). APCI is utilized less frequently in plant lipid research, yet it offers distinct advantages for specific applications, e.g., for prenylquinone analysis (Martinis et al. 2011).

In many laboratories, liquid chromatography (LC) is employed for sample delivery to the MS instrument. This method reduces matrix effects and can resolve isomers. A notable disadvantage of LC–MS is the increased analysis time when compared to DI-MS analyses. However, advancements in ultra-performance liquid chromatography (UPLC) systems with pressure limits up to 1000–1500 bar have made it easier to overcome this limitation, because they enable short run times along with increasing resolution of lipids on LC columns with small particle sizes (1.7–2 µm). The most commonly used LC phases are reversed phase (RP), while normal phase (NP) and hydrophilic-interaction phases (HILIC) are less common (Jouhet et al. 2017).

In comparison to LC separation, DI-MS analyses are faster and therefore more suitable for high-throughput and comprehensive lipid profiling (Welti et al. 2002; Han and Gross 2005; Ejsing et al. 2009). The most relevant limitations are ion suppression of low abundant or non-polar lipids (which can be addressed through the utilization of TLC or solid-phase extraction (SPE) enrichment as previously outlined), and isobaric interference. However, high-resolution instruments (QTOF, Orbitrap) are capable of resolving ions with highly similar mass-to-charge ratios.

## Nomenclature and standardization

**Fig. 1:**
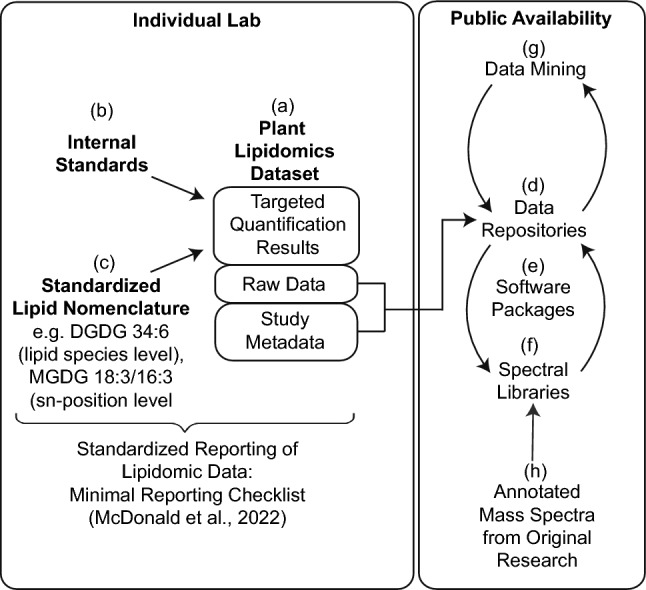
Strategies for handling plant lipidomic datasets. **a** Individual labs use their analytical workflow to measure lipids and generate lipidomic datasets, **b** documenting their use of internal standards and other analytical details in a form to meet FAIR criteria as described in the minimum reporting checklist. **c** Quantitative (or relative) data are reported using a standardized nomenclature for complex lipids, including the details available through the chosen analytical technique (McDonald et al. 2022). **d** The raw data along with metadata of the study can be made publicly available via open data repositories. **e** The original researcher can employ software tools (e.g., MS-Dial) connected to spectral libraries (**f**) to complement the targeted analysis and find additional, initially missed or falsely-annotated lipids. **g** For the scientific community, raw data uploaded to data repositories can be reprocessed for other non-intended studies, which increases the potential scope of each uploaded dataset. Other researchers can search lipidomic data in the repositories using specific queries and perform meta-analyses. Spectral libraries (**f**) can continuously be optimized for plant lipid research by adding curated or community-rated spectra (**h**)

Standardized lipid annotation is critical for the reproducibility of data in plant lipidomic experiments (Fig. 1). However, the efforts made by lipidomic experts to harmonize lipid nomenclature did not consider a significant number of plant lipids (Fahy et al. 2005). The updated version of the LIPID MAPS nomenclature now encompasses the most prevalent plant-specific lipids, including plastidial glycoglycerolipids (mono-, digalactosyldiacylglycerol, MGDG, DGDG) and unusual lipids (e.g., sphingolipids, very-long-chain fatty acids, sterol lipids) (Liebisch et al. 2020). This nomenclature facilitates hierarchical reporting of lipid species (e.g., MGDG 34:6), *sn* positions (e.g., MGDG 18:3/16:3), and double-bond localization (e.g., MGDG 18:3(9*Z*,12*Z*,15*Z*)/16:3 (2*E*,4*E*,6*E*), resulting in an enhanced transparency and comparability of the datasets. A significant proportion of plant lipidomic datasets has been recorded in Arabidopsis, and a substantial body of evidence exists to support the structural characteristics of lipids, including *sn* positions of glycerolipids and the double-bond positions of acyl groups, based on gas chromatography (GC) and TLC data (Welti et al. 2002). Consequently, a simplified nomenclature of Arabidopsis lipids with regard to structural resolution at the lipid species level (e.g., MGDG 36:6) is frequently employed for high-throughput studies.

## Targeted plant lipidomics

The focus of targeted plant lipidomic studies is frequently on the changes in lipids during periods of stress or during different developmental stages, with a particular emphasis on model species such as Arabidopsis and crop plants. Targeted plant lipidomic techniques involve the analysis of predefined lipid classes and molecular species, predominantly of plant lipids that have been structurally elucidated. The analysis employs meticulously optimized lipid extraction, chromatographic separation, and MS data acquisition. A high-throughput workflow for the quantification of the major phosphoglycerolipids and galactoglycerolipids using DI-MS with multiple reaction monitoring on QQQ instruments has been established, and this method has been employed in a large number of studies (Welti et al. 2002). The identification of molecular species is contingent upon MS2 experiments and a scan for lipid class-selective fragments after collision-induced dissociation, for instance the neutral loss of a galactose moiety for MGDG. Other labs have developed similar quantification strategies, often employing LC separation coupled to a QQQ instrument. Further strategies have been established for the quantification of sphingolipids and sterol lipids (Markham et al. 2006; Wewer et al. 2011). A significant amount of effort has been dedicated to the analysis of GIPCs, the most abundant sphingolipid class in plants. Lipidomic studies on GIPCs have revealed a high level of complexity of polar headgroups, which is similar to that observed in gangliosides in animal cells (Markham et al. 2006; Buré et al. 2014; Yu et al. 2023). The quantification of GIPCs is constrained by the availability of internal standards. Recent efforts in plant lipidomics have focused on the harmonization of methodologies, encompassing the use of standards, with the objective to increase comparability of published datasets (https://lipidomicssociety.org/interest_groups/plant-and-algal-lipids/) (McDonald et al. 2022).

## Standards for mass spectrometry of lipids

Quantification strategies rely on internal standards to correct for factors such as extraction efficiency, ion suppression, and molecular species‑dependent signal responses (Fig. 1). Theoretically, absolute quantification at the molecular species level is only feasible when authentic standards exist for each analyte; otherwise, semi‑quantitative or class‑normalized reporting is more common. The limited availability of commercial standards optimized for plant lipids (e.g., stable-isotope labeled standards or plant lipids with odd acyl chain lengths) has resulted in a heterogeneity of internal standards employed by plant lipid laboratories. The laboratory of Ruth Welti has established a comprehensive set of internal standards, comprising both commercially available and in-house modified (hydrogenated) compounds, for the most abundant membrane lipid classes. This set is widely utilized by other laboratories (Shiva et al. 2018). Plant glycerolipids are characterized by their high degree of unsaturation (e.g., containing 18:3 and 16:3 acyl groups). Consequently, the utilization of unsaturated lipid standards is recommended. In recent developments, deuterated and unsaturated plant lipid standards have become available from companies such as Avanti Polar Lipids. These standards serve as examples of appropriate reference material. Additional challenges in plant lipidomics are posed by matrix effects derived from complex plant extracts, particularly due to components such as chlorophyll, pigments, and non-polar lipids.

## Non-targeted plant lipidomics

Non-targeted workflows have been developed to go beyond targeted analyses of known lipids, by the identification of differentially accumulating lipids or lipid patterns in studies on plant stress, development, various ecotypes, and other biological questions. The use of a high-resolution MS instrument is imperative for non-targeted lipidomics to ensure a high coverage of identified molecular features and lipids. Consequently, in contrast to targeted workflows (which predominantly rely on LC-QQQ instruments), non-targeted analyses typically employ high-resolution MS instruments (QTOF, Orbitrap) with LC (or UPLC) separation prior to ionization. A limitation of UPLC is the narrow peak sizes of only a few seconds (few data scans possible) in combination with the relatively slow scan speed of QTOF and Orbitrap instruments (ca. 10–100 scans/s) as compared to QQQ (ca. 100–> 1000 scans/s). A solution that has been implemented by a number of instrument vendors is a data-independent analysis/tandem MS mode (e.g., SWATH/Sciex, Q-RAI/Agilent), in which all ions within a predefined *m*/*z* window are systematically fragmented and analyzed. This mode facilitates a comprehensive and at the same time unbiased detection of lipids. More than 200 lipids were recently mapped across various Arabidopsis tissues, employing a sophisticated LC–MS/MS approach with data-independent analysis. Thereby, tissue-specific lipid remodeling processes were unveiled (Kehelpannala et al. 2021). These data were then incorporated into an electronic fluorescent pictograph (eFP) browser (see below). Another dimension of resolution is provided by the ion mobility technology that separates molecular ions in a gas-filled compartment based on their steric characteristics. This technology can be advantageous in the separation of isomeric and isobaric compounds. The ion mobility method has been successfully applied to the analysis of plant lipids (Shvartsburg et al. 2011), yet it is expected that further studies on plant lipids using ion mobility techniques will be conducted in the future (Joshi et al. 2024). In addition to the aforementioned high-resolution techniques, the identification of a maximal number of molecular features/lipids with non-targeted analyses relies on the availability of reference mass spectra from experimental and in silico spectral libraries (Table 2).

**Table 2:** Overview on selected data repositories and their use in plant lipidomics

Repository	Access	Suitability for plant lipidomics	Link
MassIVE (UCSD)	Open	Suited for plant lipidomics, yet few datasets uploaded	https://massive.ucsd.edu/ProteoSAFe/static/massive.jsp?redirect=auth
MetaboLights (EMBL-EBI)	Open	Suited for plant lipidomics, yet few datasets uploaded	https://www.ebi.ac.uk/metabolights/
Metabolomics Workbench (NIH)	Open	Suited for plant lipidomics, yet few datasets uploaded	https://www.metabolomicsworkbench.org/
Zenodo	Open	Suited for plant lipidomics, yet few datasets uploaded, designed to accommodate very large datasets	https://zenodo.org/

## Data repositories and spectral libraries for plant lipids

Data repositories are utilized for the purpose of storing raw and processed experimental datasets (Fig. 1). These datasets include full MS runs, along with the relevant metadata, sample descriptions, methods, etc. A plethora of data repositories for plant metabolomic datasets are available, encompassing both commercial (vendor-specific) and free data banks (Table 2). However, these repositories contain few datasets on plant lipidomics. In case that datasets are made available to the public by authors in a repository, these datasets will be accessible to other researchers for the purpose of reprocessing and further mining. This additional work may often extend beyond the research scope of the original study. To search mass spectral libraries, the language package MassQL (Mass Spectrometry Query Language) has been developed for the purpose of translating mass spectral features into text. It is a powerful (vendor- and instrument-independent) tool that can be used to search for specific lipid patterns in large datasets from an individual research group as well as in previously published datasets (Damiani et al. 2025).

Different software packages are distributed by instrument vendors (commercial) or research institutes (e.g., MetaboAnalyst, XCMS, MS-Dial). The purpose of these software tools is to process original raw data, perform peak picking, and link data to spectral libraries for molecular feature annotation. Commercial tools provided by instrument vendors are usually limited in terms of the use of data formats, because they are typically confined to those that are vendor-specific. In contrast, non-commercial tools frequently offer a more extensive array of data formats and are often available without costs. Additional tools, including mzmine, have been developed with the purpose of enhancing the process of peak picking. These tools offer further options which are not accessible in commercial software packages.

Spectral libraries are repositories of reference spectra (typically MS/MS) that are associated with a particular compound entry (name, formula, structure, class). These libraries are utilized for the identification of unknown molecular features through the process of mass spectral matching. For the compound entries of lipids, the short-hand notation is widely employed (Liebisch et al. 2020). However, the list of plant lipids is not yet comprehensive, and the coverage of plant lipids in the spectral libraries needs improvement. Depending on the specific library, the mass spectra may be derived from experimental data (from standards, well-annotated samples or from community shared data including LIPID MAPS, Metlin/XCMS, MassBank, GNPS/MassIVE), based on in silico data (LipidBlast, MS2Lipid) or from both (Kind et al. 2013; Sakamoto et al. 2024). The quality of lipid annotation is contingent on the quality of the entries in the spectral libraries. It is evident that the coverage of plant lipids in these libraries is currently limited. The scientific community has made significant contributions to the enhancement of spectral libraries by providing additional datasets. The meticulous curation of these libraries also plays a crucial role in ensuring the reliability of the information they contain. These efforts will collectively lead to a substantial improvement in the quality of lipid annotation.

## Imaging of tissue distribution and subcellular localization of lipids

The analysis of lipids by DI-MS or LC–MS necessitates the isolation of lipid extracts from whole tissues or organ samples. However, it is not possible to determine the lipid composition on a cellular or subcellular level. Consequently, MS methods have been developed to achieve spatial resolution of lipid patterns in plants. For instance, a limited number of cells can be collected using laser-capture microdissection technology for subsequent analysis in genomics, transcriptomics, and proteomics experiments. While previous methods of fixation and embedding of the specimen in chemical polymers were usually not compatible with subsequent lipid measurements by MS, samples obtained by cryofixation followed by laser-capture microdissection can be used for DI-MS approaches (Knittelfelder et al. 2018).

The advent of MS imaging (MSI) techniques has facilitated the localization of lipids at the cellular level. MSI was utilized to ascertain the distribution of membrane and storage lipids in leaves and seeds (Horn and Chapman 2024). In the majority of MSI techniques, the molecules are ionized through the use of a laser beam (MALDI). Consequently, the spatial resolution of MALDI-MSI is restrained by the raster step size and the diameter of the laser beam. The resolution is typically in the range of 10 µm, but even subcellular resolutions of 3–5 µm have been reported (Sturtevant et al. 2016). An alternative MSI technique is DESI which does not necessitate a chemical matrix. Rather, it is based on desorption in liquid droplets generated with a charged solvent. DESI-MSI is associated with a spatial resolution of 50 µm or higher. The establishment of MALDI-MSI instruments with narrower diameter of the laser beam is required to determine the lipid distribution on a subcellular level, which is in the µm to sub-µm range.

Confocal laser scanning fluorescence microscopy (CLSM) represents an alternative technique for the subcellular localization of lipids. The resolution of CLSM has seen significant enhancement in recent decades, to the extent that observation of individual molecules has become feasible. For the localization of small molecules, such as lipids, two techniques can in principle be employed: (i) the use of antibodies which bind to a specific lipid in the membranes of plant cells presented in a thin section (immunofluorescence microscopy). The primary antibody is visualized by CLSM after binding to a secondary antibody which is covalently linked to a fluorophore. For instance, antiserum raised against the galactolipid DGDG was utilized to localize DGDG in plastidial and mitochondrial membranes following P deprivation (Jouhet et al. 2004). (ii) Biosensor proteins represent another tool for the subcellular localization of small molecules including lipids. In a so-called ‘translocation sensor’, a specific lipid binding domain is fused to a fluorescent protein. Upon binding to the cognate lipid, the lipid-binding domain undergoes a conformational change which is transduced to the fluorescent protein domain, resulting in the alteration of the emission wavelength which is recorded by CLSM. Most biosensor proteins used in plant lipid research are derived from animal/human sequences. The first biosensors in plants were targeted at phosphoinositide lipids (Simon et al. 2014). Additional biosensors specific for phosphatidic acid (PA), diacylglycerol (DG), and phosphatidylserine (PS) have been developed later (Colin et al. 2022). Biosensors specific for the major phosphoglycerolipids phosphatidylcholine (PC) and phosphatidylethanolamine (PE), for glycoglycerolipids, or for sphingolipids are still missing. The establishment of additional biosensors holds great potential to localize the different lipids on a subcellular level, in different cell types, and under various stress conditions in the future.

## Studying unusual lipids using mass spectrometry

**Fig. 2:**
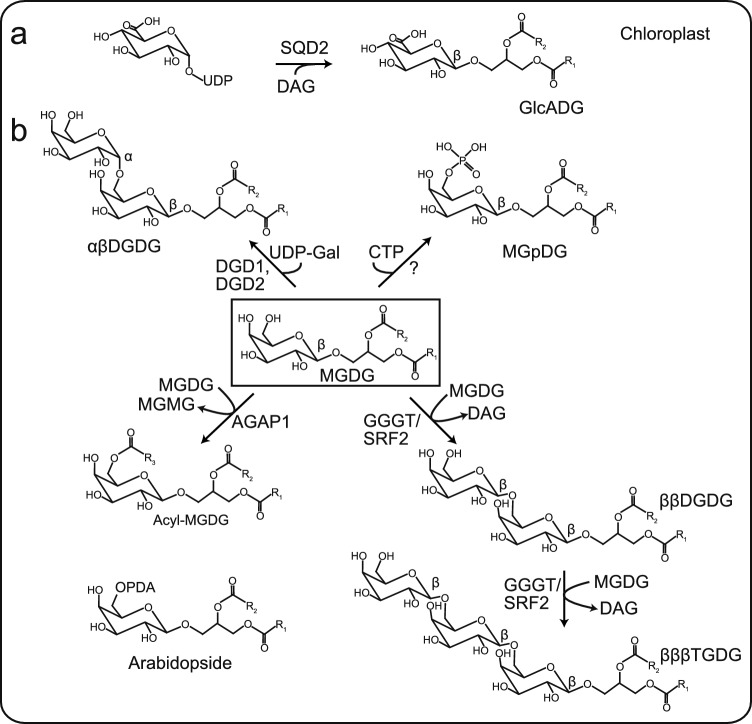
Synthesis of unusual glycolipids in the chloroplast. **a** Synthesis of glucuronosyldiacylglycerol (GlcADG) by SQD2 in the chloroplast. **b** MGDG is the precursor for different unusual glycoglycerolipids. In the canonical galactolipid synthesis pathway, αβDGDG is produced by DGD1 or DGD2. The recently discovered phospholipase AGAP1 transfers an acyl group from one MGDG to the C6 hydroxyl group of another MGDG to produce acyl-MGDG. Note that it is unknown whether the headgroup-bound acyl group is oxidized to OPDA, or whether the oxidation occurs first and the oxidized acyl group is transferred to the galactose moiety (Arabidopsides). The GGGT/SFR2 enzyme transfers a galactose moiety from MGDG to another MGDG or ββDGDG, resulting in ββDGDG or βββTGDG synthesis. MGDG can be phosphorylated at the haedgroup (MGpDG) by an unknown kinase. *AGAP1* acylated galactolipid associated phospholipase 1, *CTP* cytidine triphosphate, *DG* diacylglycerol, *DGD1, DGD2* digalactosyldiacylglycerol synthase 1, 2, *DGDG* digalactosyldiacylglycerol, *GGGT* galactolipid:galactolipid galactosyltransferase, *GlcADG* glucuronosyldiacylglycerol, *MGDG* monogalyctosyldiacylglycerol, *MGpDG* monophosphogalactosyldiacylglycerol, *MGMG* monogalactosylmonoacylglycerol/lyso-MGDG, *ODPA* oxophytodienoic acid, *SFR2* sensitive to freezing 2, *SQD2* sulfolipid synthase, *TGDG* trigalactosyldiacylglycerol, *UDP-Gal* uridine diphosphate-galactose

It is widely acknowledged that the majority of lipids and lipid biosynthetic pathways have been characterized in plants such as Arabidopsis. A plethora of novel discoveries have been made in the last 2 decades, and we herein present several examples from the metabolism of plastidial glycoglycerolipids. The structural characterization by modern MS methods has contributed to our understanding of the synthesis and function of unusual glycoglycerolipids in plastids which accumulate in low amounts compared with the abundant galactolipids MGDG and DGDG. During periods of freezing stress, oligogalactolipids like trigalactosyldiacylglycerol (TGDG) accumulate in chloroplasts (Heemskerk et al. 1985). The corresponding enzyme involved in TGDG synthesis (galactolipid:galactolipid galactosyltransferase, GGGT) was identified in 2010 (Fig. 2) (Moellering et al. 2010). GGGT is identical to SFR2 (sensitive to freezing 2), a glycosyl hydrolase which transfers a galactose from one MGDG to another MGDG or DGDG, producing DGDG or TGDG, respectively. GGGT/SFR2 is activated under freezing stress and thus contributes to the plant’s adaptation to low temperature. In contrast to ‘normal’ galactolipids like DGDG which carry the first galactose bound in β-glycosidic linkage and the second one in α-glycosidic linkage, all galactose moieties in GGGT/SFR2-derived oligogalactolipids are bound in β-glycosidic linkage. The separation and identification of αβDGDG and ββDGDG were achieved by LC–MS analysis on a HILIC column (Okazaki et al. 2013b).

It has been known for some time that plants change their membrane lipid composition under P deprivation by replacing phosphoglycerolipids with non-phosphorous glycoglycerolipids, including DGDG and sulfoquinovosyl diacylglycerol (SQDG). The sulfolipid SQDG is essential to replace the anionic phosphoglycerolipid phosphatidylglycerol (PG) in the chloroplast. During SQDG synthesis in Arabidopsis, UDP-sulfoquinovose is first produced from UDP-glucose and sulfite by SQD1, and the sulfoquinovose moiety is transferred from UDP-sulfoquinovose to diacylglycerol (DG) by the sulfolipid synthase SQD2 (Pugh et al. 1995; Benning et al. 2008). In 2013, Okazaki and co-workers employed a non-targeted approach to find the novel glycoglycerolipid glucuronosyldiacylglycerol (GlcADG) in chloroplasts of Arabidopsis under P deprivation (Fig. 2) (Okazaki et al. 2013a). GlcADG is synthesized by headgroup transfer from UDP-glucuronic acid catalyzed by SQD2, and it represents another anionic lipid in addition to SQDG that replaces phosphatidylglycerol (PG) under P deprivation.

Another class of plastidial glycoglycerolipids is represented by galactolipids carrying a third fatty acid bound to the C6 hydroxyl group of the galactose moiety in MGDG or DGDG. Acyl-MGDG and acyl-DGDG were discovered more than 50 years ago (Fig. 2) (Heinz and Tulloch 1969). Other acylated galactolipids are the so-called Arabidopsides E and G, which include galactolipids carrying an oxylipin fatty acid, e.g., oxophytodienoic acid (OPDA), bound to the C6 hydroxyl group of the headgroup. Acylated galactolipids accumulate during wounding, freezing, and bacterial infection, as shown by DI-MS analyses (Song et al. 2021). The gene encoding the corresponding acyltransferase designated ‘acylated galactolipid associated phospholipase 1’ (AGAP1) was discovered in 2015 (Nilsson et al. 2015). Expression of AGAP1 is induced under freezing stress and during the hypersensitive response.

In 2000, another headgroup modification of the galactolipid MGDG was described, because whole chloroplasts or chloroplast membranes accumulated MGDG carrying a radioactive phosphate group on the galactose (monophosphogalactosyldiacylglycerol, MGpDG) after incubation with ^32^P-labeled CTP (Fig. 2) (Müller et al. 2000). This result suggests that the envelope membranes harbor an unknown CTP-dependent kinase which phosphorylates the headgroup of MGDG. While phosphorylated molecular species of MGDG have not yet been detected in MS analyses, the corresponding kinase and the function of phosphorylated MGDG in chloroplasts remain enigmatic.

## A comprehensive database for plant lipid research

MS-based plant lipidomics has transformed our understanding of lipid diversity and dynamics in plants. While targeted workflows provide quantitative insights into known lipids, non-targeted approaches are instrumental to uncover novel lipid species, driven by advances in high-resolution MS, spectral libraries, and data-independent acquisition. However, challenges persist in terms of standardization, extraction efficiency, and the coverage of understudied lipids. Nonetheless, collaborative efforts facilitate the establishment of more robust and reproducible plant lipidomic workflows. Future directions in this field include the development of techniques for spatial resolution, the integration of Artificial Intelligence (AI), and the expansion of spectral libraries to fully capture the complexity of plant lipidomes.

With the growing amount of data on plant lipid research, it is becoming increasingly challenging to provide a comprehensive overview of the structures, occurrence, pathways, and functions of the various lipid classes and molecular species (Fig. 1). A number of databases have been developed for the purpose of storing data on plant lipid research, including LIPID MAPS, LipidBank, SwissLipid, PlantFAdb (Table 3). Additional databases focus on lipidomic results or on plant genomic or transcriptomic data in Arabidopsis or other plant species. It must be noted that each database has its limitations, and there is currently no database that contains a comprehensive collection of lipid data. The concept of a ‘virtual plant’ as a model for growth and development has been proposed. The virtual plant software platform connects parameters of growth and development with genomic, transcriptomic, proteomic, and metabolomic data (Katari et al. 2010). The ideal virtual plant database would provide comprehensive data on an organ, tissue, and single cell level for various growth conditions and developmental stages, extending to the model plant Arabidopsis and potentially other (crop) species. To achieve a comprehensive understanding of the subject, it is important that data pertaining to the subcellular distribution of lipids and pathways is incorporated into the database. A sophisticated database presenting lipid data on the plant tissue level was established by incorporating lipid data into an eFP browser (https://bar.utoronto.ca/efp_arabidopsis_lipid/cgi-bin/efpWeb.cgi). This approach facilitates the integration of gene expression data with lipid profiles for the examined Arabidopsis tissues and developmental stages (Kehelpannala et al. 2021). Such platforms would be highly valuable for the understanding of the biosynthesis and function of the substantial number of lipids and their molecular species in plants in the future.

**Table 3:** Overview on selected spectral libraries and their use for plant lipidomics

Resource	Access	Content/coverage/limitations	Link
GNPS community spectral libraries	Open	Large community MS/MS libraries from standards, extracts, plant lipids, plant natural products; mostly ESI MS/MS spectra, few EI (GC–MS) spectra	https://gnps.ucsd.edu/ProteoSAFe/static/gnps-splash.jsp?redirect=auth
Golm Metabolome Database	Open	Open library, many plant metabolites and lipids, limited to EI (GC–MS) spectra	http://gmd.mpimp-golm.mpg.de/
LIPID MAPS	Open	Large library with (ESI) MS/MS spectra of lipid standards, few plant-specific lipids	https://www.lipidmaps.org/
LIPID_BANK_	Open	MS/MS, NMR, and UV spectra of natural lipids with structures, includes plant lipids, often references to relevant literature	https://lipidbank.jp/
LipidBlast	Open	Large in silico MS/MS library, therefore limitations in applicability; focus on lipids, few plant-specific lipids	https://fiehnlab.ucdavis.edu/projects/lipidblast
MassBank	Open	Open mass spectral library for small molecules, experimental (ESI) MS/MS and EI records, many lipid spectra, few plant-specific lipids	https://massbank.eu/MassBank/ https://mona.fiehnlab.ucdavis.edu/ https://www.massbank.jp/MassBank/
MetaboLights (EMBL-EBI)	Open	Compound library with structures and MS/MS spectra; many lipid spectra, few plant-specific lipid spectra (but featured in the compound list)	https://www.ebi.ac.uk/metabolights/
METLIN	Open^1^	Large library of (ESI) MS/MS spectra from standards, available through XCMS; many lipids, few plant lipids	https://metlin.scripps.edu/auth-login.html
mzCloud	Commercial (subscription)	High-quality MS/MS with advanced tools; many MS/MS spectra of lipid standards, few plant-specific lipids	https://www.mzcloud.org
NIST/EPA/ NIH Mass Spectral Library	Commercial	Commercially curated MS libraries, mostly electron impact (EI) spectra; not specific for lipids, good coverage of non-complex plant lipids (fatty acids, sterols)	https://sciencesolutions.wiley.com/solutions/technique/gc-ms/nist-epa-nih-mass-spectral-library/
PlantFAdb	Open	Plant fatty acid structures, metabolic network; not a spectral library; comprehensive plant fatty acid database with references	https://fatplants.net/plantfadb/ based on the Seed Oil Fatty Acid (SOFA) database
ReSpect (RIKEN)	Open	MS^n^ spectral database for phytochemicals*,* plant metabolites, but few plant lipids; references to respective literature	http://spectra.psc.riken.jp
SwissLipids	Open	Curated library of lipid structures, metabolic network and details of biological roles with references to studies; many plant lipids	https://beta.sparql.swisslipids.org/

These repositories contain electrospray ionization (ESI) spectra if not indicated otherwise

## Data Availability

Not applicable.

## Abbreviations

DESI: Desorption electrospray ionization
DGDG: Digalactosyldiacylglycerol
DI-MS: Direct infusion-mass spectrometry
MGDG: Monogalactosyldiacylglycerol
GIPC: Glycosylinositol phosphorylceramide
MALDI: Matrix-assisted laser desorption/ionization
LC–MS: Liquid chromatography–mass spectrometry
QQQ: Triple quadrupole
QTOF: Quadrupole time of flight
TGDG: Trigalactosyldiacylglycerol
TLC: Thin-layer chromatography
UPLC: Ultra-performance liquid chromatography
X:Y: Fatty acids
X: Number of carbons
Y: Number of double bonds
